# Enrichment of human Vγ9Vδ2 T lymphocytes by magnetic poly(divinylbenzene-*co*-glycidyl methacrylate) colloidal particles conjugated with specific antibody

**DOI:** 10.1039/c8ra01468j

**Published:** 2018-04-17

**Authors:** Piamsiri Sawaisorn, Tienrat Tangchaikeeree, Duangporn Polpanich, Panuwat Midoeng, Rachanee Udomsangpetch, Abdelhamid Elaissari, Kulachart Jangpatarapongsa

**Affiliations:** Center for Research and Innovation, Faculty of Medical Technology, Mahidol University Bangkok 10700 Thailand kulachart.jan@mahidol.ac.th; National Nanotechnology Center, National Science and Technology Development Agency (NSTDA), Thailand Science Park Pathum Thani 12120 Thailand; Department of Pathology, Army Institute of Pathology, Phramongkutklao Hospital Bangkok 10700 Thailand; University Lyon-1, CNRS, LAGEP UMR 5007 43 Boulevard du 11 Novembre 1918 69100 Villeurbanne France

## Abstract

γδ T cells play a significant role in protection against cancer. Purification of γδ T cells is needed for insight when studying their anti-cancer functionality and their utilization in adoptive cell therapy. To improve the purification of γδ T cells, in this work, a composite material based on magnetic nanoparticles was developed for purification of Vγ9Vδ2 T cells, the predominant subset of γδ T lymphocytes in human peripheral blood. The epoxy-functionalized magnetic poly(divinylbenzene-*co*-glycidyl methacrylate) particles (mPDGs) were bio-conjugated with anti-human Vδ2 antibody to provide specific recognition sites for T cell receptors of Vγ9Vδ2 T cells. Using fluorescence-activated cell sorting (FACS) analysis, separation of Vγ9Vδ2 T cells from peripheral blood mononuclear cells of healthy donors was confirmed with high purity [89.77% (range 87.00–91.80, *n* = 3)]. More interestingly, the immobilized particles did not affect the viability of purified cells as high cell viability was indicated (>90%). By combining the properties of magnetic nanoparticles with specific antibodies, these immobilized particles were shown to be used as a cell-friendly purification tool of Vγ9Vδ2 T lymphocytes without any limits for the further use of cells. The purified Vγ9Vδ2 T cells using the antibody-immobilized epoxy-functionalized mPDGs could be used directly without a detachment step for further cultivation and expansion. This highlights the advantages of this method in allowing the study of cell function and further investigation of such rare T cell populations in immunotherapy.

## Abstract 2

Schematic procedure of Vγ9Vδ2 T cell purification using antibody-immobilized epoxy-functionalized mPDGs.

## Graphical abstract

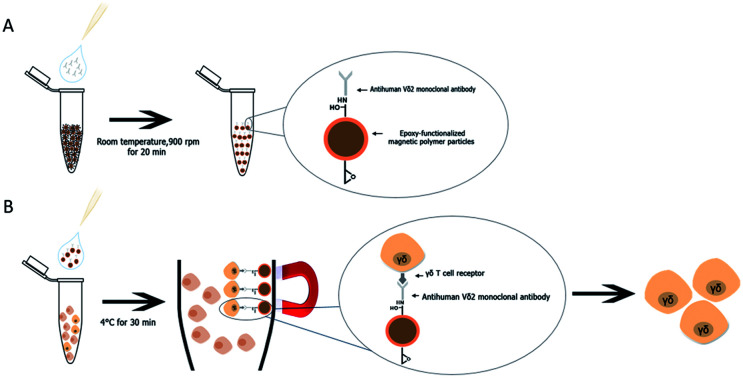

## Introduction

T lymphocytes are classified into two subsets, αβ T cells and γδ T cells, based on their T cell receptors (TCRs). These receptors have been characterized using monoclonal anti-TCR antibodies (mAb) and methods to identify gene rearrangement of cytotoxic T lymphocytes.^1–4^ αβ T cells play an important role in adaptive immune responses, mostly in regard to their peptide antigen recognition in the context of major histocompatibility complex (MHC) class II or class I molecules and in the production of effector molecules.^5^ γδ T cells are unconventional T lymphocytes that express TCRs consisting of heterodimeric γ and δ chain complexes for antigen recognition.^6^ In normal human peripheral blood, most γδ T cells are defined by their expression of the variable domains Vγ9 and Vδ2 of the TCR and are referred to as Vγ9Vδ2 T cells, and represent only 1–10% of total T cells.^7^ These cells show unique features with antigen recognition, which is unrestricted to MHC molecules, and TCR gene usage.^8^ As a result, Vγ9Vδ2 T cells act as immune surveillance cells which respond rapidly with respect to pathogens, stressed- or infected-cells and cytokines.^9^ Because of their capacity to infiltrate tumors and then express cytotoxic activity, these cells are also involved in anti-cancer immune responses.^10,11^ Moreover, Vγ9Vδ2 T cells also have been shown to display the principal characteristics of professional antigen-presenting cells.^12^

Based on the attractive characteristics of Vγ9Vδ2 T cells, several clinical trials were initiated to purify these cells from peripheral blood for further study. In addition, the culture of Vγ9Vδ2 T cells was developed in order to obtain high numbers of cells for adoptive cell transfer in cancer treatment.^13^ Utilizing their nonpeptide, phosphoantigen recognition ability,^14^ this cell population can be expanded *in vitro* using phosphoantigens such as bromohydrin pyrophosphate (BrHPP) or nitrogen-containing bisphosphonates (N-BPs), and can be purified for use in clinical treatments.^15,16^ Recent clinical trials have included approaches where magnetic labeling systems and column isolation were used for selection of purified TCR γδ-expressing T cells. By using separation kits, the purity of these cells is more than 90% after separation from peripheral blood mononuclear cells (PBMCs).^17–19^ However, the process is complex and time-consuming. Moreover, long incubation time may lead to non-specific cell labeling. To address this problem various functionalized magnetic polymer nanoparticles, such as magnetic nanoparticles modified with hydrazine functionalized polymer, fluorescent chitosan functionalized magnetic polymeric nanoparticles and epoxy-functionalized magnetic poly(divinylbenzene-*co*-glycidyl methacrylate) particles (mPDGs), were utilized.^20–22^ By reason of their properties, along with highly specific surface areas and versatile surface functionality, these particles can be used widely as carriers for biomolecules including proteins, antibodies and antigens.^23–25^ Moreover, a great advantage of using magnetic nanoparticles is the rapidity of separation upon applying an external magnetic field, a one-step process which can avoid many of the time-consuming steps of other separation processes.^26^ With the usefulness of mPDGs, there is current report with respect to using the immobilized mPDGs with monoclonal anti-human IL-10 antibody to obtain specific and selective recognition sites for the recombinant human IL-10 protein in an immunoassay.^25^ In addition, these mPDGs were successfully applied for purification of CD4^+^ T lymphocytes suggesting that mPDGs could be a good candidate for Vγ9Vδ2 T cell separation.^27^

In this study, in order to improve the efficiency of Vγ9Vδ2 T cells isolation, we aimed to investigate the properties of epoxy-functionalized mPDGs intended for use in the *in vitro* separation and purification of Vγ9Vδ2 T cells. The immobilized magnetic particles were first bioconjugated with monoclonal anti-human Vδ2 antibody to provide specific recognition of the TCR Vδ2 presented on Vγ9Vδ2 T cells. To determine percentage of purity and viability of Vγ9Vδ2 T cells, conjugation with fluorescence antibodies which recognized specific surface markers of Vγ9Vδ2 T cells was performed followed by fluorescence-activated cell sorting (FACS) analysis.

## Experimental

### Epoxy-functionalized mPDGs preparation

The epoxy-functionalized mPDGs were synthesized following a previously published method.^25^ Briefly, seed emulsion copolymerization of divinylbenzene (DVB, 1 mL) (Sigma, USA) and glycidyl methacrylate (GMA, 0.1 mL) (Sigma, USA) monomers was conducted using potassium persulfate (KPS, 0.05 g) (Sigma, USA) as an initiator and in the presence of O/W magnetic emulsion droplets (1.4% w/v) as a seed (kindly provided by Mohamed M. Eissa from University Lyon-1, France). The polymerization reaction was carried out at 70 °C while stirring (300 rpm) and under N_2_ atmosphere for 24 h. The obtained mPDGs were filtered using glass wool fibers to remove any coarse particles before further analyses.

Hydrodynamic size and zeta potential of the prepared particles were measured using Zetasizer (Malvern, Nano ZS2000). In addition, a transmission electron microscope (TEM) (Phillips, CM120) and vibrating sample magnetometer (NETZSCH, TG209) were utilized for studies of morphological and magnetic content.

### Antibody immobilization onto epoxy-functionalized mPDGs

The specific anti-human Vδ2 antibody (mouse IgG1k, clone B6; BioLegend, USA) was immobilized onto the surface of epoxy-functionalized mPDGs. In brief, 1 mg of epoxy-functionalized mPDGs (2.2% w/v) were washed twice with PBS buffer (pH 7.4, 500 μL). After that, the purified anti-human Vδ2 antibody (20 μg mL^−1^) was added to the particles and incubated at room temperature for 20 min using a ThermoMixer (Eppendorf, Germany). After centrifugation, the supernatant was collected to determine the residual concentration of antibody at 260 nm using a UV-Vis spectrophotometer (Thermo Scientific, USA). The binding efficiency was calculated using the following equations.

1 Binding efficiency = [(*C*_i_ − *C*_f_)/*C*_i_] × 100

where *C*_i_ and *C*_f_ (mg mL^−1^) are the initial and final concentrations of anti-human Vδ2 antibody. The antibody-immobilized particles were washed twice with PBS buffer (pH 7.4) and the supernatant was finally discarded. The non-specific binding of contaminants was blocked with PBS containing bovine serum albumin (0.1% w/v) and sodium azide (0.05% w/v). The immobilized particles were stored at 4 °C until used.

### Preparation of peripheral blood mononuclear cells (PBMCs)

Whole blood from healthy volunteers was collected at the blood bank unit of Phramongkutklao Hospital. Buffy coats from whole blood (50–70 mL) were collected in blood bags containing anticoagulant for further isolation of PBMCs. Collected buffy coats were overlaid on Lymphoprep™ solution (Axis Shield PoC, Oslo, Norway) at a 1 : 1 ratio by volume and centrifuged (Hettich Zentrifugen, Germany) at 2000 rpm and 20 °C for 30 min. The interface layer containing the PBMCs was collected and washed twice with cold incomplete RPMI 1640 medium (Gibco, USA) containing 10 000 U mL^−1^ penicillin–streptomycin (Gibco, USA). The PBMCs were suspended in cold PBS (100 μL) and kept at 4 °C prior to use. Viability of isolated cells was assessed using the trypan blue exclusion method. Obtaining all buffy coats from human donations at the blood bank of Phramongkutklao Hospital followed an informed consent process approved by Mahidol University Ethics Committee, Mahidol University, Bangkok, Thailand (MURA2014/400).

### Generation of human Vγ9Vδ2 T cells from peripheral blood mononuclear cells

To cultivate human Vγ9Vδ2 T lymphocytes, total PBMCs isolated from buffy coats by density gradient centrifugation using Lymphoprep™ were resuspended in RPMI 1640 supplemented with FBS (10% w/v) and penicillin–streptomycin (10 000 U mL^−1^) in the presence of pamidronate (10 μM) and recombinant human IL-2 (50 IU mL^−1^ final concentration). The PBMCs were plated on 24-well plates with addition of IL-2 every 3 days. After 12 days of culture, the cells were examined to determine their percentage and viability by flow cytometric analysis (BD FACSCANTO II, BD Biosciences, USA) and trypan blue exclusion, respectively. The cells in cultures containing high percentages of Vγ9Vδ2 T cells were harvested and suspended in cold PBS (100 μL) and kept at 4 °C before starting isolation experiments.

### Separation of human Vγ9Vδ2 T cells by antibody-immobilized epoxy-functionalized mPDGs from peripheral blood

PBMC samples from healthy donors were processed to separate Vγ9Vδ2 T cells using antibody-immobilized epoxy-functionalized mPDGs. The separation conditions were optimized by varying the amount of antibody-immobilized particles (20, 100 or 200 μg) and the amount of PBMCs (1 × 10^6^, 5 × 10^6^ or 10 × 10^6^ cells). Briefly, PBMCs at a certain concentration in cold PBS (100 μL) were mixed with 20, 100 or 200 μg of antibody-immobilized epoxy-functionalized mPDGs. The mixtures were incubated at 4 °C for 15, 30 or 60 min, gently stirred, and then complexes were separated by applying a magnet. The percentage and viability of Vγ9Vδ2 T cells were determined using FACS analysis and trypan blue exclusion, respectively.

### Separation of human Vγ9Vδ2 T cells by antibody-immobilized mPDGs from Vγ9Vδ2 T cells cultivation system

The optimal conditions determined with PBMCs and antibody-immobilized mPDGs were used to separate Vγ9Vδ2 T cells from cell cultures. The percentages of Vγ9Vδ2 T cells were investigated by FACS analysis and viabilities were determined by trypan blue exclusion. To compare the result, separation of Vγ9Vδ2 T cells was also done by negative selection using TCRγ/δ^+^ T Cell Isolation Kit and immunomagnetic sorting (Miltenyi Biotec, Germany) according to the manufacturer's instructions.

### Immunophenotyping by flow cytometry

Purity of the separated Vγ9Vδ2 T cells was determined within 1 × 10^5^ PBMCs using FACS analysis, before and after isolation using antibody-immobilized epoxy-functionalized mPDGs. The percentage of Vγ9Vδ2 T cells were determined by analyzing the TCR including Vγ9 and Vδ2 (Vγ9^+^Vδ2^+^). The cells were incubated at 4 °C for 15 min with a combination of fluorescent-labeled antibodies as follows to determine the percentages of subsets of Vγ9Vδ2 T cells: anti-CD3-PE-cy5 (HIT3a), anti-CD8-PE-cy7 (HIT8a), anti-CD4-PEXTR (13B8.2), anti-TCR Vγ9-PE (B3) and anti-TCR Vδ2-FITC (B6) (BioLegend, USA) and analyzed by FACS.

### Statistical analyses

Statistical significance was determined by one-way analysis of variance (ANOVA). One-way ANOVA with Bonferroni's multiple comparison test was used to assess the significance of differences between varying amounts of antibody-immobilized epoxy-functionalized mPDGs and incubation times. One-way ANOVA with Dunnett's multiple comparison test was used to compare between groups and controls. In addition, a two-tailed, paired *t* test was used to assess the significance of differences between groups. All of the tests utilized GraphPad Prism version 5.01 (GraphPad Software). In bar graphs, results are expressed as mean ± SEM. *P* values < 0.05 were considered statistically significant.

## Results and discussion

### Characterizations of mPDGs

**Fig. 1:**
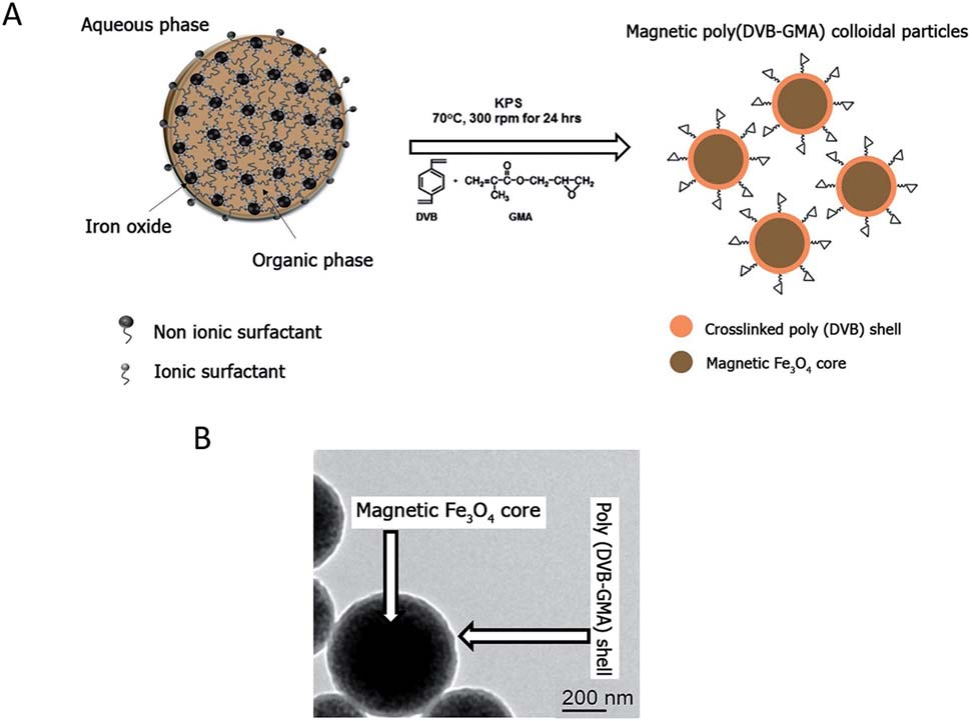
(A) Schematic procedure of mPDG preparation. (B) TEM micrographic analysis of the mPDGs.

Reactive epoxy-functionalized mPDGs were successfully developed following the published method of Eissa and colleagues.^25^ Seed emulsion co-polymerization of DVB and GMA monomers in the presence of Fe_3_O_4_ magnetic emulsion droplets as the seed was elaborated. The hydrodynamic size of the prepared functionalized mPDGs was 488 nm, zeta potential at pH 7.3 was −59 mV and the magnetic content was 67% wt. From TEM, the obtained mPDGs exhibited a dark magnetic core and well-defined polymer shell (as shown in Fig. 1).

To date, interest in magnetic nanoparticles or functional polymer hybrid materials has increased greatly within the biomedical field. Functionalized magnetic polymer nanoparticles can be used not only for *in vivo* therapeutic applications,^28^ but also for *in vitro* applications including the separation and purification of biomolecules such as proteins and nucleic acids.^29–32^ Their surface functionality make them suitable for wide use as carriers of antibodies which detect specific antigens.^33,34^ There are two approaches used for bio-conjugated between the surface of the nanoparticle and the antibody, physical adsorption and direct covalent linkage.^35^ The covalent binding is currently preferred because the strength of interaction between biomolecule (*i.e.*, chemical properties of the antibodies) and the reactive particle (*i.e.*, surface dispersion) can be manipulated. Moreover, such interaction can prevent an unexpected competitive displacement of the adsorbed antibodies by other blood components.^36^ The major advantage of using magnetic polymeric nanoparticles is their easy manipulation by applying a magnet to the material and the resulting rapidity of separation. For these particles, magnetite (Fe_3_O_4_) is most frequently used as the magnetic core. This iron oxide is superparamagnetic, easily prepared, and biocompatible when utilized in biomedical applications.^37^

### Immobilization of anti-human Vδ2 antibody onto epoxy-functionalized mPDGs

Anti-human Vδ2 antibody was covalently bound to the epoxy-functionalized mPDGs by using appropriate conditions with no need of a coupling agent. The binding efficiency of antibody-immobilized mPDGs of 70.97% (range 58.35–96.65, *n* = 5) was calculated by using eqn (1). The results, therefore, indicated that most anti-human Vδ2 antibody was immobilized on the particle surface.

As mentioned previously, we chose the GMA monomer as a precursor to produce reactive functional polymers. GMA contains a polymerizable double bond and a reactive epoxy group allowing it to be chemically grafted with any biomolecule which contains reactive functional groups without using a coupling agent.^24^ This enabled us to covalently attach the selected antibodies *via* their functional groups to the particle surface. Our result is in line with previous reports.^25,38^ The reactive epoxy-functionalized mPDGs were easily bio-conjugated with the anti-human Vδ2 antibody and the covalent binding process leads to a strong conjugation which is suitable for various types of biologic application. Moreover, the binding efficiency can be further elevated by varying the amount of antibodies or the incubation time of the interaction.

### Separation of human Vγ9Vδ2 T cells by antibody-immobilized epoxy-functionalized mPDGs

**Fig. 2:**
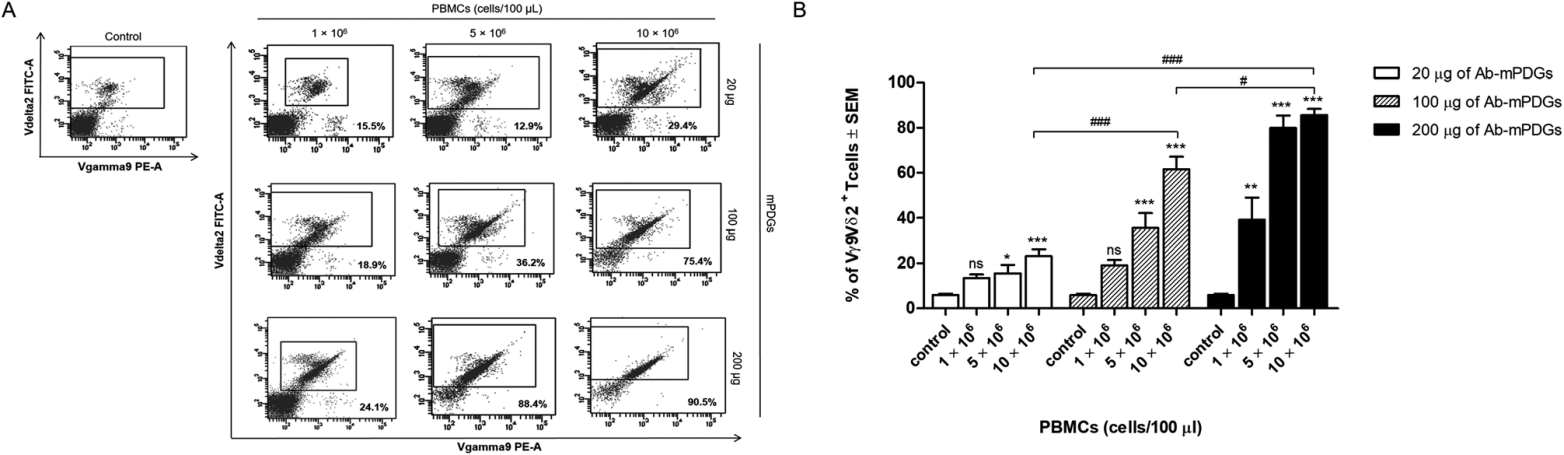
Separation of Vγ9Vδ2 T cells by using antibody-immobilized mPDGs. Finding the optimal condition for separation of Vγ9Vδ2 T cells was done by varying the amount of antibody-immobilized mPDGs and PBMCs. The cells were stained with the specific antibodies, anti-TCR Vγ9-PE and anti-TCR Vδ2-FITC, and analyzed by flow cytometry before and after isolation. (A) Data shown are representative flow cytometry panels with the purity of the Vγ9Vδ2 T cells before and after purification. Numbers in each panel indicate the purity of Vγ9Vδ2 T cells. (B) Purity of Vγ9Vδ2 T cells before (control) and after separation by varying the amount of antibody-immobilized mPDGs and PBMCs are shown as mean ± SEM from 5 independent experiments performed with different healthy donors. *p < 0.05, **p < 0.05, ***p < 0.0001, ^#^*p* = 0.0354, ^###^*p* < 0.0001; one-way ANOVA.

The antibody-immobilized epoxy-functionalized mPDGs were used for separation of human Vγ9Vδ2 T cells from peripheral blood of healthy donors. In order to determine optimal conditions, the amount of antibody-immobilized mPDGs (20, 100 or 200 μg), number of PBMCs (1 × 10^6^, 5 × 10^6^ or 10 × 10^6^ cells), and incubation time (15, 30 or 60 min) were varied. It was found that the purity of separation was significantly higher when there were 10 × 10^6^ PBMCs suspended, irrespective of the amount of antibody-immobilized particles, when compared to the lower numbers of PBMCs (as shown in Fig. 2).

It was noticed that at 10 × 10^6^ PBMCs, the highest purity (mean = 85.60%) of Vγ9Vδ2 T cells was obtained with the use of 200 μg of antibody-immobilized epoxy-functionalized mPDGs (*p* < 0.001). Decreases in the amount of the particles (20 and 100 μg) were associated with the purity of the separated Vγ9Vδ2 T cells, all less than 85.60% (as shown in Fig. 2).

**Fig. 3:**
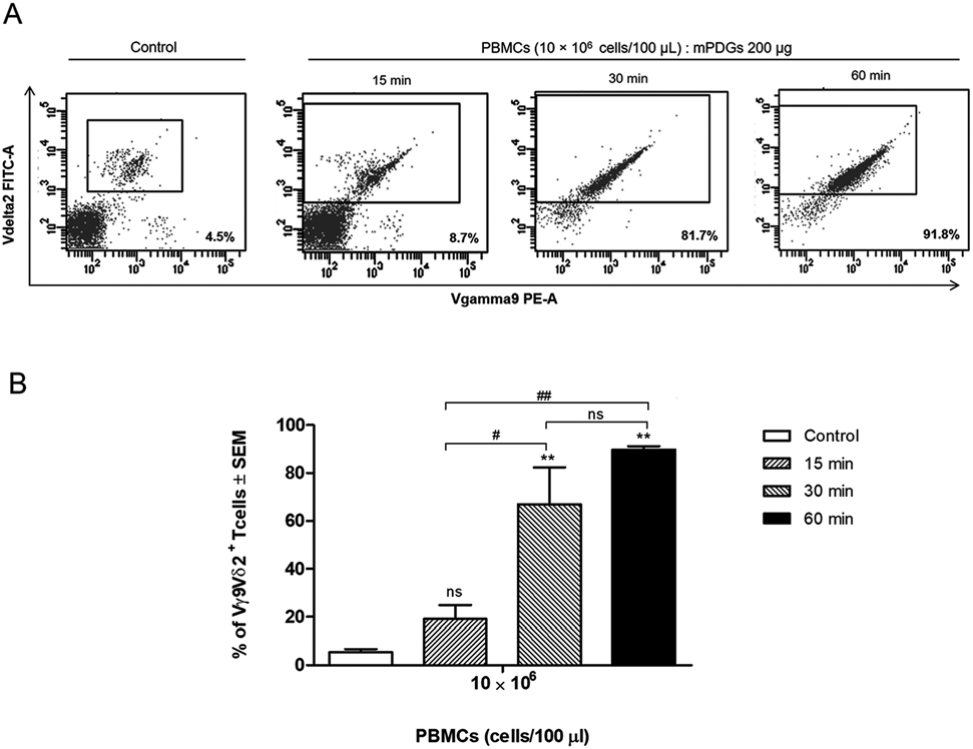
Optimization of incubation time for separation of Vγ9Vδ2 T cells by using antibody-immobilized mPDGs. (A) Representative flow cytometry panels with the Vγ9Vδ2 T cells before and after purification. Numbers in each panel indicate the purity of Vγ9Vδ2 T cells. (B) Purity of Vγ9Vδ2 T cells before (control) and after separation by varying incubation time. Data shown are mean ± SEM of one representative of 3 experiments performed by using PBMC samples from different healthy donors. **p = 0.0014, ^##^*p* = 0.0014, ^#^*p* < 0.05; one-way ANOVA.

Regarding variation of incubation time, it was shown that at 30 min of co-incubation the separation reaction of Vγ9Vδ2 T cells was started and that the purity of the separated Vγ9Vδ2 T cells was significantly greater (*p* = 0.0014) than that of controls (mean = 66.97%) (Fig. 3). The results also showed that there was no significant difference in the purity of separation between incubation times of 30 and 60 min, although the highest purity was seen at 60 min. Of note, the anti-human Vδ2 antibody used possessed high specificity for Vγ9Vδ2 T cells. The optimal conditions for separation of human Vγ9Vδ2 T cells by immobilized epoxy-functionalized mPDG was the use of 10 × 10^6^ PBMCs with 200 μg antibody-immobilized mPDGs and 30 min of incubation time.

The efficiency of mPDGs for *in vitro* cell separation was investigated by isolating the rare Vγ9Vδ2 T cells. This subset of T cells has raised interest from researchers in the last decade because of their distinctive surface molecules and the antigen-binding characteristics leading to their immune-patrolling capacity. Their effector function (initiation of immune responses) upon infectious pathogens is described elsewhere.^7,9^ Normally, Vγ9Vδ2 T cells are a minor population in human peripheral blood representing only 1–10% of the total T cells.^7^ Several trials have attempted to achieve pure populations of these cells. The separation techniques commonly applied to this small cell population include both positive and negative selection. Both selection methods are based on magnetic microbead labeling and column separation using different antibody labeling of target cells. For positive selection, the target cells are obtained through the direct binding of anti-TCR γ/δ hapten-antibody labelling and anti-hapten microbeads along with the magnetic isolation.^19^ This method has proved successful for many researchers and often yields greater than 90% purity of the Vγ9Vδ2 T cells. However, these separation processes are time-consuming and confounded by contamination with dead cells and non-labeled cells caused by long incubation times.

To overcome this, we generated a simple protocol for Vγ9Vδ2 T cell purification. This is the first evidence showing that antibody-immobilized epoxy-functionalized mPDGs can be used for separation of the rare Vγ9Vδ2 T cell population from PBMCs with high levels of purity. Our protocol includes few steps and allows easy and rapid specific separation of the targeted cells. Furthermore, under the effect of an external magnetic field, this present method was feasible and rapid.

### Separation of human Vγ9Vδ2 T cells by antibody-immobilized epoxy-functionalized mPDGs from Vγ9Vδ2 T-cell cultivation system

**Fig. 4:**
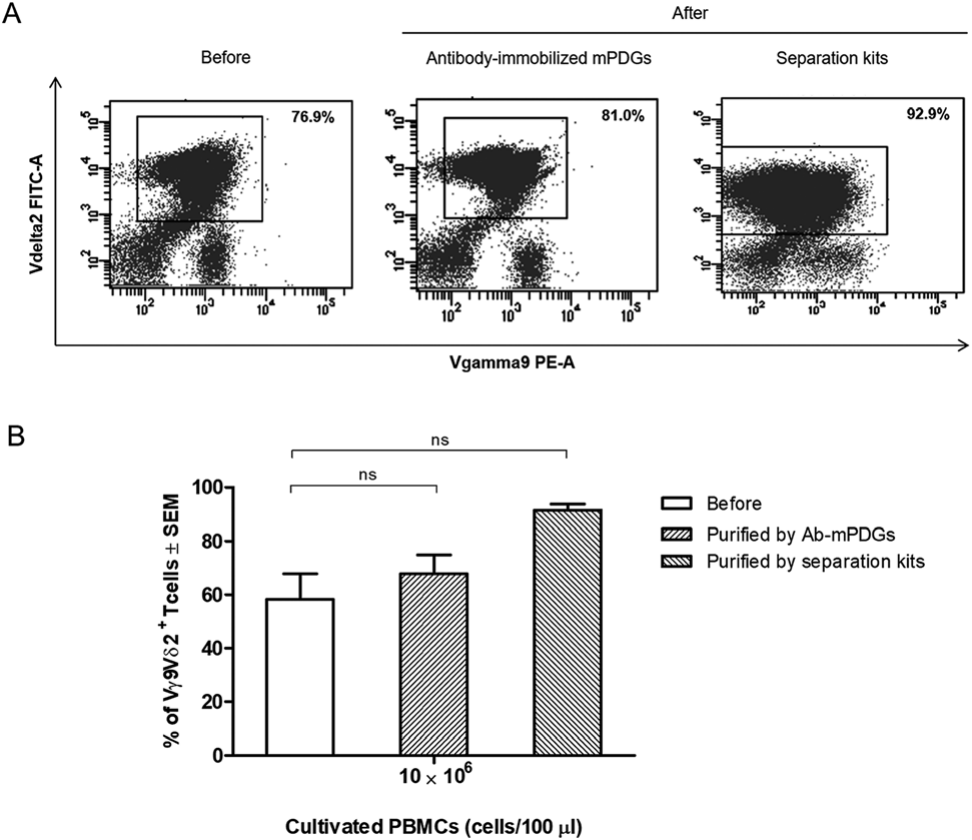
Separation of Vγ9Vδ2 T cells from a Vγ9Vδ2 T-cell culture system by using antibody-immobilized mPDGs and separation kits. *In vitro*-cultured Vγ9Vδ2 T cells were separated and purified. The cells were stained with specific antibodies, anti-TCR Vγ9-PE and anti-TCR Vδ2-FITC, and analyzed by flow cytometry. (A) Data shown are representative flow cytometry panels with the purity noted of the Vγ9Vδ2 T cells before and after purification. (B) Purity of Vγ9Vδ2 T cells before and after separation by using antibody-immobilized mPDGs and separation kits are shown as mean ± SEM from different 3 experiments carried out in duplicate.

In cancer immunotherapy using adoptive T cell transfer, Vγ9Vδ2 T cells are a critical factor in obtaining favorable results because of their specific binding to small non-peptide antigens derived from various cancer cell lines.^10^ A protocol to obtain pure populations and heighten the yield of Vγ9Vδ2 T cells is important for achieving the best outcomes with γδ T-cell immunotherapy. Synthetic phosphoantigens are utilized in cell stimulation to gain high amounts of γδ T cells, together with purification steps as shown previously.^39,40^ Seeking to improve the outcome of the separation step, the antibody-immobilized epoxy-functionalized mPDGs were also used for separation of human Vγ9Vδ2 T cells from *in vitro* Vγ9Vδ2 T-cell cultures. The efficiency of the separation of Vγ9Vδ2 T cells was evaluated by flow cytometry. The results from FACS analyses revealed that the purity of Vγ9Vδ2 T cells was 67.88% (as shown in Fig. 4).

However, the amount of obtained Vγ9Vδ2 T cells did not shift significantly indicating that the antibody-immobilized particles could be used for both cell sources, but that a greater purification might be reached when used with peripheral blood as shown in our optimal conditions determination, which provide evidence that the obtained amount of human Vγ9Vδ2 T cells could be reached to 89.77% performed by using PBMC samples (Fig. 3). Hence, to increase purity, the optimization using antibody-immobilized epoxy-functionalized mPDGs for further used in Vγ9Vδ2 T cell separation from cultivation source may be important.

**Fig. 5:**
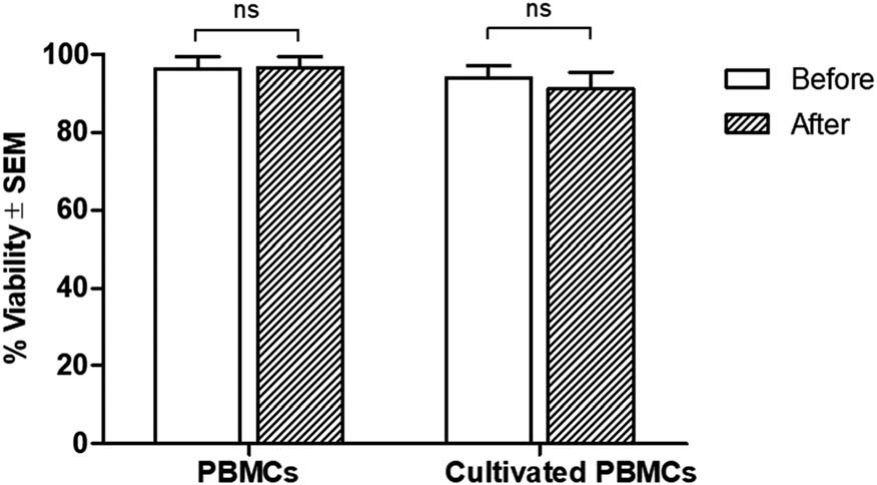
Viability of Vγ9Vδ2 T cells purified by using antibody-immobilized mPDGs. Viability was determined by trypan blue exclusion of cells before and after separation. Data shown are the mean values of viability ± SEM from one representative experiment of 3 independent experiments performed with different PBMC samples and Vγ9Vδ2 T cells lines.

Regarding cell viability after separation, results from the trypan blue exclusion tests are shown in Fig. 5. It was found that antibody-immobilized epoxy-functionalized mPDGs did not alter the viability of separated cells, indicating that this protocol was useful for the purification of Vγ9Vδ2 T cells and would allow further study of cellular function. Furthermore, mPDGs which are the synthetic nanoparticles with the presence of magnetite (Fe_3_O_4_) as a core and polymer shell are preferred in biomedical applications because they are biocompatible^25^ and nontoxic as shown in the result. Therefore, it may be suggested that mPDGs do not have to be removed for downstream application such as cell culture.

Regarding the small size of mPDGs, they could be phagocytosed by antigen presenting cells including macrophages and dendritic cells which are crucial cells for adaptive immune system activation by phagocytosis.^41,42^ B cells, a type of lymphocytes, constitute only 1–7% in peripheral blood also function as antigen presenting cells.^43,44^ This did not effect the T cells separation as the high purity (mean = 85.60%) of Vγ9Vδ2 T cells was found with the use of highest amount of mPDGs (Fig. 2).

We showed in this study that in-house, antibody-immobilized epoxy-functionalized mPDGs could be used to purify Vγ9Vδ2 T cells from an *in vitro* cultivation system using pamidronate, a specific activator for expansion of this T cell subtype.^45^ However, a few contaminants from other cell populations occurred during the purification, suggesting the importance of further refinement of the protocol. But most noteworthy, these antibody-immobilized epoxy-functionalized mPDGs were nontoxic to the cells which was also confirmed by previous study.^27^ This developed isolation tool is expected to be employed for providing the ease way in adoptive T cell transfer using Vγ9Vδ2 T cells which imparts a great benefit to the immunological and immunotherapy studies of Vγ9Vδ2 T cells as important immune effector cells.

## Conclusions

**Fig. 6:**
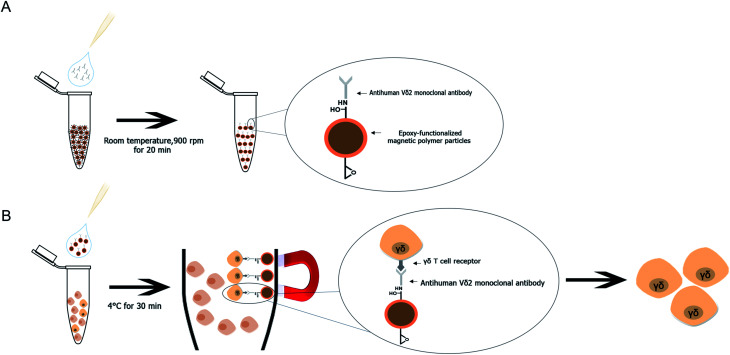
Schematic procedure of Vγ9Vδ2 T cell purification using antibody-immobilized epoxy-functionalized mPDGs. (A) Immobilization of anti-human Vδ2 antibody onto epoxy-functionalized magnetic particles. (B) Capture of the Vγ9Vδ2 T cells by the antibody-immobilized particles and enrichment of the target cells.

A method for Vγ9Vδ2 T cell separation by using purified human Vδ2 antibody immobilized mPDGs (antibody-immobilized mPDGs) was developed as a useful tool in various research fields (as shown in Fig. 6). The specific antibody was immobilized onto epoxy-functionalized mPDGs. When co-incubation with PBMCs or *in vitro* Vγ9Vδ2 T cell cultures, the interaction between monoclonal antibody-immobilized particles and Vδ2 T cell receptor (TCR) of Vγ9Vδ2 T cells could be performed, allowing excellent separation. Due to the ease of processing and short incubation time, the use of antibody-immobilized epoxy-functionalized mPDGs appears attractive for the preparation of Vγ9Vδ2 T cells. The advantage of using specific antibody was to separate the Vγ9Vδ2 T cells with good viability, allowing further functional studies which could help to better understand their roles in cancer biology and immunotherapy. This technique could potentially be used in various research studies and could be adapted for the separation of other cells of interest.

## Conflicts of interest

There are no conflicts to declare.

## Supplementary Material
